# New Efficient Synthesis of 3,4-Dihydropyrimidin-2(1*H*)-ones Catalyzed by Benzotriazolium-Based Ionic Liquids under Solvent-Free Conditions

**DOI:** 10.3390/molecules21040462

**Published:** 2016-04-07

**Authors:** Zhiqing Liu, Rong Ma, Dawei Cao, Chenjiang Liu

**Affiliations:** 1 Key Laboratory of Oil and Gas Fine Chemicals of Ministry of Education, School of Chemistry and Chemical Engineering, Xinjiang University, Urumqi 830046, China; 15293606480@163.com (Z.L.); marong19851208@126.com (R.M.); lzxlxx123@126.com (D.C.); 2 Physics and Chemistry Detecting Center, Xinjiang University, Urumqi 830046, China; pxylcj@126.com

**Keywords:** Benzotriazolium-based ionic liquids, Biginelli reaction, synthesis, catalysis

## Abstract

An efficient synthesis of novel 3,4-dihydropyrimidin-2(1*H*)-ones (DHPMs) and their derivatives, using Brønsted acidic ionic liquid [C_2_O_2_BBTA][TFA] as a catalyst, from the condensation of aryl aldehyde, β-ketoester and urea was described. Reactions proceeded smoothly for 40 min under solvent-free conditions and gave the desirable products with good to excellent yields (up to 99%). The catalyst could be easily recycled and reused with similar eﬃcacies for at least six cycles.

## 1. Introduction

In recent years, 3,4-dihydropyrimidin-2(1*H*)-ones (DHPMs) and their derivatives have received much attention because they are important substructures in both biologically active compounds and several marine alkaloids involving the DHPM core units [1]. A simple and direct approach for their synthesis involves the conjugate addition of aryl aldehyde, β-ketoester and urea in the presence of either protic or Lewis acids. In recent years, several improved methods have been reported for the preparation of these compounds using various catalysts such as *p*-TsOH·H_2_O [2], H_3_BO_3_ [3], [Al(H_2_O)_6_](BF_4_)_3_ [4], thiamine hydrochloride [5], l-(+)-tartaric acid-dimethylurea [6], imidazole-1–yl-acetic acid [7], HClO_4_-SiO_2_ [8], polyvinylsulfonic acid [9], SnCl_2_·2H_2_O [10], NaCl [11], SrCl_2_·6H_2_O [12], Al-planted MCM-41 [13], (NH_4_)_2_CO_3_ [14], CeCl_3_·7H_2_O [15], CaCl_2_ [16], Ce(NH_4_)_2_(NO_3_)_6_ [17] and Fe(OTs)_3_·6H_2_O [18]. However, several of these reported procedures suffer from some drawbacks such as strong acidic conditions, long reaction times, use of expensive or hazardous reagents, complex handling and low yields of products. Moreover, most of these methods employ organic solvents as the reaction medium. Hence, new, efficient and environmentally friendly procedures are still strongly demanded in organic transformations such as condensation reactions.

**Scheme 1:**
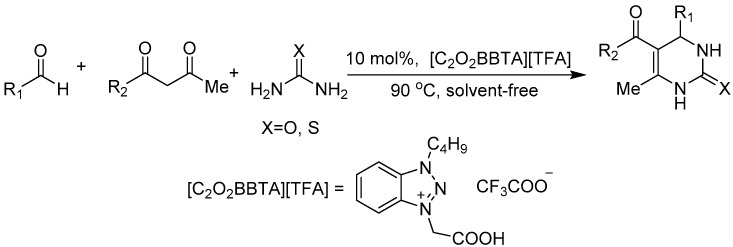
Condensation of aryl aldehyde, β-ketoester and urea in the presence of [C_2_O_2_BBTA][TFA].

Currently, ionic liquids (ILs) have been widely used as environmentally benign reaction media and catalysts in organic synthesis owing to their unique properties of non-volatility, excellent solubility, high thermal stability and recyclability [19,20]. In particular, the synthesis of task-specific ILs (TSIL) with special functions according to the requirement of a specific reaction has become an attractive field. Extensive effort has been focused on the elucidation of the mechanism of Lewis acid–catalyzed Biginelli reactions in ionic liquids [21]. Sharma *et al.* [22] reported highly recyclable amino acid ionic liquids as a catalyst, particularly glycine nitrate, for the one-pot, three-component Biginelli condensation under microwave irradiation (MW). Recently, Kandasamy and co-workers realized the synthesis of 1-alkyl triazolium triﬂate room temperature ionic liquids and their catalytic studies in a multi-component Biginelli reaction [23]. In continuation of our interest in the Biginelli reaction [24,25,26], herein we employ Brønsted acid ionic liquid 1-butyl-3-carboxymethyl-benzotriazolium trifluoroacetate [C_2_O_2_BBTA][TFA] as a catalyst to study the possibility of synthesizing DHPMs under solvent-free conditions (Scheme 1).

## 2. Results and Discussion

The catalytic activity of [C_2_O_2_BBTA][TFA] was investigated in a one-pot Biginelli condensation of aryl aldehyde, β-ketoester and urea. The results are presented in Table 1. The best result was achieved by carrying out the reaction at 90 °C for 40 min in the presence of 10% catalytic amount of [C_2_O_2_BBTA][TFA] without any solvent (Table 1, entry **8**). Inspired by Clark’s work [27], we explored the relationship between the catalyst and solvents (Table 1, entries **1**–**8**). When molecular solvents, such as H_2_O, MeOH, CH_3_CN or toluene, were employed, the reaction afforded a mixture of benzaldehyde, ethyl acetoacetate and urea under similar conditions, and DHPMs were obtained only in a very low yield (<19%). When no catalyst was used in this reaction system, the reaction did not give the desired product. This showed that ionic liquid plays a very important role in the reaction system (Table 1, entry **9**). The influence of the reaction time on the yield was also investigated as shown in Table 1, entries **8**, 15–19. It turned out that although the reaction time was increased to 40 min, the yield did not change significantly (Table 1, entry **8**). For the purpose of saving energy, we chose 40 min as the reaction time. Hence, the best conditions employed a 0.1:2:2:3 mole ratio of [C_2_O_2_BBTA][TFA], aryl aldehyde, β-ketoester, and urea at 90 °C for 40 min under solvent-free conditions.

The recycling performance of TSIL [C_2_O_2_BBTA][TFA] was one of its most important benefits, which was also investigated in the reaction of aryl aldehyde, β-ketoester and urea. After separation of the product, the filtrate containing catalyst was vacuumed to remove water and the resulting catalyst was reused directly for the next run. As shown in Table 1, Brønsted acidic ionic liquid [C_2_O_2_BBTA][TFA] can be recycled at least six times without showing a significant decrease in catalytic activity, and the yields ranged from 96% to 92% (Table 1, entry **8**). This indicated that ionic liquid [C_2_O_2_BBTA][TFA] was an eﬃcient and recyclable catalyst for the preparation of 3,4-dihydropyrimidin-2(1*H*)-ones derivatives.

In order to explore the scope and limitations of this reaction, we extended the procedure to various aryl-substituted aldehydes carrying either electron-donating or -withdrawing groups in the *ortho*, *meta*, and *para* positions. In general, the reaction proceeded easily under the best conditions and the adducts were isolated in excellent yields and high purity. In addition, compared to the reported synthetic method of 6-methyl-2-oxo-4-phenyl-1,2,3,4-tetrahydropyrimidine-5-carboxylate (Table 2, entry **3a**) by using HCl as a catalyst and ethonal as a solvent [28], our strategy has the advantages of higher yield (96% *vs.* 78%) and shorter reaction time (40 min *vs.* 3 h). The obtained results indicated that the electron-donating or -withdrawing groups at the aryl ring did not seem to affect the reaction significantly in terms of yield (Table 2, entries **3a**–**3o**). Thiourea has been used with similar success to provide the corresponding *S*-dihydropyrimidinones analogues, which are also of interest due to their biological activities (Table 2, entries **3p**–**3t**). The use of different substituted β-ketoester as a 1,3-dicarbonyl moiety in place of ethyl acetoacetate also gave similar results, as shown in Table 2 (entries **3u**–**3ab**).

## 3. Experimental Section

All melting points were determined using a Büchi B-540 instrument. All melting points are uncorrected. All new compounds were characterized by IR, ^1^H- and ^13^C-NMR spectra. The IR spectra were obtained as potassium bromide pellets with a FTS-40 spectrometer (BIO-RAD, Hercules, CA, USA). The ^1^H-NMR spectra were measured on a Varian Inova-400 spectrometer (at 400 and 100 MHz, respectively) using TMS as an internal standard in CDCl_3_ or DMSO-*d*_6_.

### 3.1. General Procedure for the Synthesis of 1-Butyl-3-carboxymethyl-benzotriazolium Trifluoroacetate

**Scheme 2:**
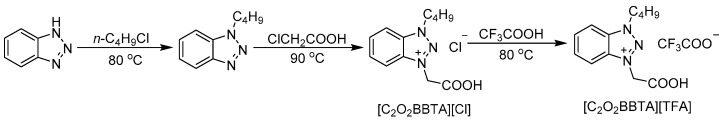
Synthesis of [C_2_O_2_BBTA][TFA].

[C_2_O_2_BBTA][TFA]: benzotriazole (0.2 mol) and chlorobutane (0.24 mol) were dissolved in 30% aqueous solution of sodium hydroxide (100 mL). Tetrabutylammonium bromide (1 g) was added and the solution was stirred 24 h at 80 °C until two phases formed. The top organic phase and bottom water phase were separated with separating funnel. Any remaining water in the organic phase was removed by decompressing Ratovapor at 70 °C [46]. The 1-butylbenzotriazole (0.1 mol) and chloroacetic acid (0.1 mol) were added to a 50 mL round bottom flask fitted with a reﬂux condenser. The solution was stirred for 36 h at 90 °C. Then the mixture was washed at least three times with diethyl ether and acetone. The product ([C_2_O_2_BBTA][Cl]) precipitated as a white solid and then was collected by filtration and dried *in vacuo* for 24 h. The [C_2_O_2_BBTA][Cl] (0.05 mol) was transferred to a 25 mL round bottom flask and trifluoroacetic acid (TFA, 0.06 mol) was added dropwise, then stirred 24 h at 80 °C. Finally, any remaining TFA was removed by decompressing Ratovapor at 90 °C for 1 h and dried *in vacuo* for 24 h (Scheme 2).

### 3.2. General Procedure for the Synthesis of 3,4-Dihydropyrimidin-2(1H)-(thio)ones

A mixture of aryl aldehyde (2 mmol), β-ketoester (2 mmol), urea (2 mmol) and [C_2_O_2_BBTA][TFA] (0.2 mmol) were heated at 90 °C under solvent-free conditions for 40 min with stirring (Scheme 1). After cooling, the reaction mixture was poured onto crushed ice (30 g) and stirred for 10 min. The separated solid was filtered under suction, washed with cold water (30 mL) and then recrystallized from ethanol to afford the pure product. The resulting precipitate was filtered under suction. The results are summarized in Table 2. All products (except **3l**–**3m**, **3y**–**3ab**) are known compounds, which were characterized by IR, ^1^H and ^13^C-NMR spectra.

*1-Butyl-3-carboxymethyl-benzotriazolium Trifluoroacetate* [C_2_O_2_BBTA][TFA]: brown liquid; ^1^H-NMR (DMSO-*d*_6_, 400 MHz, TMS): δ 0.93 (t, 3H, CH_3_), 1.31–1.41 (m, 2H, CH_2_), 2.00–2.07 (m, 2H, CH_2_), 5.11 (t, 2H, CH_2_), 6.13 (s, 2H, CH_2_), 8.00–8.53 (m, 4H, Ar-H); ^13^C-NMR (DMSO-*d*_6_, 100 MHz, ppm): δ 166.4, 158.3 (q, COCF_3_), 135.0, 134.3, 131.4, 130.9, 115.6 (q, CF_3_), 114.1, 114.0, 51.9, 51.2, 30.2, 18.7, 13.0 ppm; IR (KBr, ν/cm^−1^): 3106, 2967, 2940, 2879, 2511, 1738, 1505, 1471, 1364, 1190, 1141, 1029, 754, 718, 643, 599; ESI-MS: *m/z* (%) = 234.1 (100) [M]^+^, 113.0 (100) [M]^−^.

*5-Ethoxycarbonyl-6-methyl-4-(2-chloro-4-fluorophenyl)-3,4-dihydropyrimidin-2(1H)-thione* (**3l**): white solid; 1H-NMR (400 MHz, DMSO-*d*_6_), δ: 1.00 (t, 3H, OCH_2_CH_3_), 2.29 (s, 3H, CH_3_ ), 3.89 (q, 2H, OCH_2_), 5.59 (s, 1H, CH), 7.20 (t, 1H, Ar-H), 7.32–7.39 (dd, 2H, Ar-H), 7.73 (s, 1H, NH), 9.30 (s, 1H, NH); ^13^C-NMR (100 MHz, DMSO-*d*_6_), δ: 13.80, 17.56, 39.07, 39.59, 50.90, 58.98, 97.61, 115.53, 131,26, 138.24, 138.27, 149.28, 151.08, 159,52, 162.98, 164.74; IR (KBr, ν/cm^−1^): 3346, 3225, 3112, 2976, 1697, 1644, 1223, 1093, 903, 805.

*5-Ethoxycarbonyl-6-methyl-4-(3-bromo-4-fluorophenyl)-3,4-dihydropyrimidin-2(1H)-thione* (**3y**): white solid; 1H-NMR (400 MHz, DMSO-*d*_6_), δ: 1.09 (t, 3H, OCH_2_CH_3_), 2.25 (s, 3H, CH_3_ ), 3.99 (q, 2H, OCH_2_), 5.15 (s, 1H, CH), 7.24–7.27 (m, 1H, Ar-H), 7.33–7.37 (t, 1H, Ar-H), 7.48–7.50 (dd, 1H, Ar-H), 7.79 (s, 1H, NH), 9.29 (s, 1H, NH); ^13^C-NMR (100 MHz, DMSO-*d*_6_), δ: 13.92, 17.72, 52.97, 59.20, 98.41, 112.13, 127.44, 131.19,142.88, 148.95, 151.66, 156.05, 158.49, 164.99; IR (KBr, ν/cm^−1^): 3342, 3203, 3100, 2984, 1702, 1658, 1232, 1099, 895, 804.

*5-Isopropoxycarbonyl-6-methyl-4-(3-methoxyphenyl)-3,4-dihydropyrimidin-2(1H)-thione* (**3z**): white solid; 1H-NMR (400 MHz, DMSO-*d*_6_), δ: 1.01 (d, 3H, CH3), 1.16 (d, 3H, CH_3_CH), 2.23 (s, 3H, CH_3_C), 3.72 (s, 3H, MeO), 4.82 (m, 1H, CHCH_3_), 5.10 (s, 1H, CH), 6.76–6.83 (m, 3H, Ar-H), 7.24 (t, H, Ar-H), 7.70 (s, 1H, NH), 9.15 (s, 1H, NH); ^13^C-NMR (100 MHz, DMSO-*d*_6_), δ: 17.60, 21.54, 53.73, 54.86, 66.24, 99.34, 112.16, 118.19, 129.37, 146.34, 148.05, 152.07, 159.07, 164.73; IR (KBr, ν/cm^−1^): 3234, 3106, 2981, 2948, 1721, 1652, 1599, 1463, 1431, 1374, 1282, 1232, 1092, 1073, 788.

*5-Isopropoxycarbonyl-6-methyl-4-(4-hydroxyphenyl)-3,4-dihydropyrimidin-2(1H)-thione* (**3z**): orange solid; 1H-NMR (400 MHz, DMSO-*d*_6_), δ: 1.00 (d, 3H, CH3), 1.15 (d, 3H, CH_3_CH), 2.22 (s, 3H, CH_3_C), 4.80 (m, 1H, CHCH_3_), 5.02 (s, 1H, CH), 6.68 (d, 2H, Ar-H), 7.02 (d, 2H, Ar-H), 9.07 (s, 1H, NH), 9.31 (s, 1H, OH); ^13^C-NMR (100 MHz, DMSO-*d*_6_), δ: 17.56, 21.39, 21.69, 53.44, 66.09, 99.91, 114.79, 114.79, 127.35, 127.35, 135.46, 147.35, 152.02, 156.39, 164.79; IR (KBr, ν/cm^−1^): 3289, 3227, 3109, 2979, 2808, 1706, 1686, 1651, 1511, 1448, 1371, 1282, 1226, 1173, 1086, 783, 680.

*5-Tert-Butoxycarbonyl-6-methyl-4-(4-fluorophenyl)-3,4-dihydropyrimidin-2(1H)-thione* (**3aa**): faint yellow solid; 1H-NMR (400 MHz, DMSO-*d*_6_), δ: 1.28 (s, 9H, (CH_3_)_3_C), 2.21 (s, 3H, CH_3_C), 5.07 (s, 1H, CH), 7.13–7.18 (m, 2H, Ar-H), 7.22–7.26 (m, 2H, Ar-H), 7.66 (s, 1H, NH), 9.09 (s, 1H, NH); ^13^C-NMR (100 MHz, DMSO-*d*_6_), δ: 17.56, 27.72, 27.72, 53.60, 79.09, 100.25, 114.92, 128.12, 128.20, 141.12, 141.15, 147.43, 151.81, 159.96, 162.38, 164.61; IR (KBr, ν/cm^−1^): 3230, 3107, 2975, 2930, 1697, 1644, 1507, 1452, 1366, 1292, 1230, 1164, 1090, 1035, 837, 798, 759, 658.

*5-Tert-Butoxycarbonyl-6-methyl-4-(3-methoxyphenyl)-3,4-dihydropyrimidin-2(1H)-thione* (**3ab**): faint yellow solid; 1H-NMR (400 MHz, DMSO-*d*_6_), δ: 1.29 (s, 9H, (CH_3_)_3_C), 2.21 (s, 3H, CH_3_C), 2.27 (s, 3H, CH_3_C), 5.07 (s, 1H, CH), 7.01–7.06 (m, 3H, Ar-H), 7.18–7.22 (t, 1H, Ar-H), 7.63 (s, 1H, NH), 9.05 (s, 1H, NH); ^13^C-NMR (100 MHz, DMSO-*d*_6_), δ: 17.56, 21.01, 27.72, 27.72, 27.72, 54.20, 78.99, 100.50, 123.22, 126.79, 127.67, 128.12, 137.07, 144.89, 147.04, 152.05, 164.73; IR (KBr, ν/cm^−1^): 3226, 3099, 2977, 2935, 1699, 1647, 1489, 1438, 1366, 1294, 1232, 1165, 1087, 859, 813, 774, 745, 697, 670, 599.

## 4. Conclusions

In summary, we have reported an efficient and convenient method for the synthesis of a series of novel dihydropyrimidin-2(1*H*)-ones using aryl aldehyde, β-ketoester and urea as substrates and employing Brønsted acidic ionic liquid [C_2_O_2_BBTA][TFA] as a catalyst. This method offers several advantages including high yields, short reaction times, and a simple work-up procedure. It also has the ability to tolerate a wide variety of substituted groups in all three components, which is lacking in existing procedures.

## Figures and Tables

**Table 1:** Effect of catalyst [C_2_O_2_BBTA][TFA] under different conditions for the reaction of aryl aldehyde, β-ketoester and urea ^a^.

Entry	Solvent	IL (mol %)	Time (min)	Yield (%) ^b^
**1**	H_2_O	10	40	5
**2**	MeOH	10	40	3
**3**	EtOH	10	40	10
**4**	CH_2_Cl_2_	10	40	16
**5**	CH_3_CN	10	40	19
**6**	DMF	10	40	NR
**7**	Toluene	10	40	5
**8 ^c^**	solvent-free	10	40	96, 95, 95, 94, 93, 92
**9**	solvent-free	None	40	NR
**10**	solvent-free	1	40	81
**11**	solvent-free	2.5	40	84
**12**	solvent-free	5	40	85
**13**	solvent-free	15	40	95
**14**	solvent-free	20	40	93
**15**	solvent-free	10	10	75
**16**	solvent-free	10	20	91
**17**	solvent-free	10	30	94
**18**	solvent-free	10	50	91
**19**	solvent-free	10	60	93

^a^ Reaction conditions: benzaldehyde (2 mmol), ethyl acetoacetate (2 mmol), urea (3 mmol) and catalyst in solvent (5 mL) or solvent-free, 90 °C; ^b^ Isolated yield; ^c^ catalyst was recycled six times.

**Table 2:** The [C_2_O_2_BBTA][TFA]-catalyzed synthesis of 3,4-dihydropyrimidin-2(1*H*)-ones ^a^.

Entry	R1	R2	X	Yields ^b^ (%)	Mp (°C) ^c^	
					Found	Reported (lit.)
**3a**	C_6_H_5_	EtO	O	96	201–202	200–202 [29]
**3b**	2-F-C_6_H_4_	EtO	O	96	236–237	235–237 [30]
**3c**	3-F-C_6_H_4_	EtO	O	97	209–211	209–211 [31]
**3d**	4-F-C_6_H_4_	EtO	O	98	175–176	175–177 [32]
**3e**	2-Cl-C_6_H_4_	EtO	O	93	211–213	211–213 [33]
**3f**	2-Br-C_6_H_4_	EtO	O	93	204–205	205–207 [30]
**3g**	3-Br-C_6_H_4_	EtO	O	94	190–191	190–192 [26]
**3h**	3-Me-C_6_H_4_	EtO	O	92	228–230	228–230 [34]
**3i**	4-Me-C_6_H_4_	EtO	O	97	209–211	209–212 [35]
**3j**	3,4-(MeO)_2_-C_6_H_3_	EtO	O	98	171–172	172–174 [36]
**3k**	3-MeO-C_6_H_4_	EtO	O	93	219–221	219–220 [37]
**3l**	2-Cl-4-F-C_6_H_3_	EtO	O	88	195–197	
**3m**	3-Br-4-F-C_6_H_3_	EtO	O	85	193–195	
**3n**	3,4-(HO)_2_-C_6_H_3_	EtO	O	89	232–234	233–235 [37]
**3o**	4-N(CH_3_)_2_-C_6_H_4_	EtO	O	89	249–251	249–250 [38]
**3p**	C_6_H_5_	EtO	S	83	202–204	202–204 [39]
**3q**	4-F-C_6_H_4_	EtO	S	86	192–193	191–192 [40]
**3r**	3-Me-C_6_H_4_	EtO	S	86	193–195	194–195 [41]
**3s**	4-Me-C_6_H_4_	EtO	S	90	184–186	185–186 [42]
**3t**	3-MeO-C_6_H_4_	EtO	S	93	140–142	141–143 [37]
**3u**	4-F-C_6_H_4_	MeO	O	98	188–189	188–190 [43]
**3v**	4-Me-C_6_H_4_	MeO	O	96	202–203	202–204 [44]
**3w**	3-MeO-C_6_H_4_	MeO	O	92	206–208	204–206 [29]
**3x**	4-OH-C_6_H_4_	MeO	O	99	231–233	231–233 [45]
**3y**	3-MeO-C_6_H_4_	*i*-PrO	O	94	196–198	
**3z**	4-OH-C_6_H_4_	*i*-PrO	O	98	192–194	
**3aa**	4-F-C_6_H_4_	*t*-BuO	O	99	147–149	
**3ab**	3-MeO-C_6_H_4_	*t*-BuO	O	95	212–214	

^a^ Reaction conditions: benzaldehyde (2 mmol), ethyl acetoacetate (2 mmol), urea (3 mmol) and catalyst in solvent-free, 90 °C; ^b^ Isolated yield; ^c^ Melting points are uncorrected.
